# Atypical diarrhoeagenic *Escherichia coli* in milk related to a large foodborne outbreak

**DOI:** 10.1017/S0950268823001395

**Published:** 2023-09-11

**Authors:** Shouhei Hirose, Kenji Ohya, Tomoya Yoshinari, Takahiro Ohnishi, Katsumi Mizukami, Tomikatsu Suzuki, Kenji Takinami, Takayoshi Suzuki, Kenichi Lee, Sunao Iyoda, Yukihiro Akeda, Yuichiro Yahata, Yuuki Tsuchihashi, Tomimasa Sunagawa, Yukiko Hara-Kudo

**Affiliations:** 1 Division of Microbiology, National Institute of Health Sciences, Kanagawa, Japan; 2 Toyama City Public Health Center, Toyama, Japan; 3 Division of Molecular Target and Gene Therapy Products, National Institute of Health Sciences, Kanagawa, Japan; 4 Department of Bacteriology I, National Institute of Infectious Diseases, Tokyo, Japan; 5 Center for Field Epidemic Intelligence, Research and Professional Development, National Institute of Infectious Diseases, Tokyo, Japan

**Keywords:** diarrhoea, *Escherichia coli* O157:H7, food poisoning, milk cartons, school lunch

## Abstract

A foodborne outbreak related to milk cartons served in school lunches occurred in June 2021, which involved more than 1,800 cases from 25 schools. The major symptoms were abdominal pain, diarrhoea, vomiting, and fever. Although major foodborne toxins and pathogens were not detected, a specific *Escherichia coli* strain, serotype OUT (OgGp9):H18, was predominantly isolated from milk samples related to the outbreak and most patients tested. The strains from milk and patient stool samples were identified as the same clone by core genome multilocus sequence typing and single-nucleotide polymorphism analysis. The strain was detected in milk samples served for two days related to the foodborne outbreak at a rate of 69.6% and levels of less than ten most probable number/100 mL but not on days unrelated to the outbreak. The acid tolerance of the strain for survival in the stomach was similar to that of enterohaemorrhagic *E. coli* O157:H7, and the same inserts in the *chu* gene cluster in the acid fitness island were genetically revealed. The pathogenicity of the strain was not clear; however, it was indicated that the causative pathogen was atypical diarrhoeagenic *E. coli* OUT (OgGp9):H18.

## Introduction

A large foodborne outbreak related to milk cartons served in school lunches occurred in June 2021 in Toyama City, Japan. A public health centre in the area first noticed that various schools such as elementary, junior high, and nursery schools reported numerous children with digestive symptoms on 17 June and began the initial epidemiological investigations [1]. The major symptoms observed in the patients were abdominal pain, diarrhoea, vomiting, and fever. Over 1,800 cases from 25 schools with no fatalities were reported. The common meals among these patients were the lunches served at each school, which included the pasteurised milk carton produced by the T milk factory. Furthermore, schools serving milk cartons produced by other factories did not report any patients. Therefore, the public health centre determined that the milk cartons produced by the T milk factory served in school lunches on 15 and 16 June were the causative food of the outbreak. T milk factory usually produced 6,000–7,000 cartons of 200 mL, 10–20 cartons of 500 mL, and 20 cartons a day from 1,600 to 1,800 kg raw milk by several workers. The raw milk was pasteurised at 128 ℃ for 2 seconds with a plate heat exchanger. The ultra-high-temperature pasteurisation method (120–150 ℃ for 2–3 seconds) is most popular in Japan, and more than 90% of milk in markets was produced by the method.

Previously, outbreaks of foodborne pathogens associated with pasteurised milk in industrialised countries have been reported [2, 3]. The major pathogens were *Salmonella*, *Listeria,* enterohaemorrhagic *Escherichia coli*, and *Yersinia enterocolitica.* A large outbreak of *Staphylococcus aureus* enterotoxins in milk products made from dried milk powder produced in a factory occurred in Japan, where more than 13,000 cases have been reported [4, 5]. These pathogens and toxins were included as targets for the investigation of the outbreak that occurred in Toyama City. Representative stool samples of 64 patients from 12 schools were tested for major foodborne pathogens as follows: *Aeromonas*, *Bacillus cereus*, enterohaemorrhagic *E. coli*, *Clostridium perfringens*, *Plesiomonas, Salmonella*, *Shigella*, *S. aureus*, *Y. enterocolitica, Yersinia pseudotuberculosis,* and norovirus, which were tested by culture methods or PCR assays in the public health centre. At a national research institute, milk samples were tested for foodborne bacterial toxins and foodborne pathogens, leading to symptoms, other than pathogens not tested in the public health centre, such as diarrhoeagenic *E. coli, Escherichia albertii,* or *Listeria monocytogenes.* In this study, we reported a microbiological analysis of a large foodborne outbreak, the suspected causative pathogen, and pathogen contamination of the milk cartons served in school lunches.

## Methods

### Milk samples

Milk carton (200 mL) samples served on 14, 15, and 16 June were stored in freezers at −20 °C in schools for investigations of food poisoning or any other accidents. Milk cartons scheduled to be served at the lunches on 17 June were stored in refrigerators. The cartons were collected by the investigators of Toyama City Public Health Center, and some of them were provided to the National Institute of Health Sciences (NIHS) for microbiological tests. The milk cartons served on 14, 15, 16, and 17 June were manufactured at the T milk factory in Toyama City, Japan, on 11, 14, 15, and 16 June, respectively.

### Detection of staphylococcal enterotoxins, B. cereus enterotoxin and the emetic toxin (cereulide), and C. perfringens enterotoxin in milk

Eleven milk samples were initially collected by Toyama City Health Center; two milk samples from two schools served on 14 June suggested no relation with the food poisoning by epidemiological investigation, two milk samples from two schools on 15 June and two milk samples from two schools on 16 June suggested the relations, and five milk samples from one school stored in the schools for lunches on 17 June but not served were tested for investigation on bacterial toxins. Additionally, five milk samples from one school stored for lunches on 17 June, but not served, were tested for the presence of bacterial toxins as described in Supplementary material.

### Detection of L. monocytogenes, S. aureus, B. cereus, E. albertii, and diarrhoeagenic E. coli in milk

The milk samples were also tested to investigate other foodborne pathogens. Because the main symptoms of patients were abdominal pain, diarrhoea, vomiting, and fever and the results of the test in Toyama City Public Health Center were referred, *L. monocytogenes*, *S. aureus, B. cereus, E. albertii,* and diarrhoeagenic *E. coli* were targeted to detect by enrichment and isolation as described in Supplementary material. In addition, *E. albertii* and diarrhoeagenic *E. coli* were tested by PCR; *E. albertii-*specific gene [6] for *E. albertii*; *stx* [7] and *eae* [8] for enterohaemorrhagic *E. coli* (EHEC); heat-labile enterotoxin (LT) [9] and heat-stable toxin (ST) [9] for enterotoxigenic *E. coli* (ETEC); *eae* [8] and *bfpA* [10] for enteropathogenic *E. coli* (EPEC); *aggR* [11] for enteroaggregative *E. coli* (EAEC); *astA* [11] for enteroaggregative *E. coli* heat-stable enterotoxin 1 (EAST1)-producing *E. coli*; and *invE* (QuickPrimer InvE gene; Takara Co., Shiga, Japan) and *ipaH* (QuickPrimer IpaH gene; Takara Co.) for enteroinvasive *E. coli* (EIEC).

### Isolation of E. coli from milk

Enrichment cultures of 11 initially collected milk samples in mEC or CT-mEC were streaked onto CHROMagar STEC (CHROMagar Microbiology, Paris, France) and DHL agar (Nissui Pharmaceutical, Tokyo, Japan) and then incubated at 37 °C for 24 h. *E. coli* colonies suspected to be mauve or blue on CHROMagar STEC and red on DHL agar were tested for biochemical characteristics using TSI (Oxoid, Hampshire, UK) and LIM (Eiken Chemical Co., Ltd., Tokyo, Japan) agars. Representative colonies showing *E. coli* biochemical characteristics were tested for O and H serotyping and genotyping, as described as follows.

Thirty-one additional milk samples (9, 10, 9, and 3 samples of 14, 15, 16, and 17 June, respectively; 25 g) were cultured in 225 mL CT-mEC at 42 °C, streaked onto CHROMagar STEC and DHL agar, and incubated at 37 °C for 24 h. Representative colonies coincident with *E. coli* biochemical characteristics were tested for O and H serotyping and genotyping as described as follows.

### Isolation of E. coli from patient faeces

Colonies suspected to be *E. coli* on DHL agar cultured from 64 patient stool samples by Toyama City Public Health Center were tested for biochemical characteristics with TSI and LIM agars. Colonies positive for *E. coli* biochemical characteristics were tested for O and H serotyping and O genotyping, as described as follows.

### O and H serotyping and O genotyping of E. coli

*E. coli* isolates were tested for *E. coli* O and H antigen agglutination with antisera according to the manufacturer’s protocols (Denka, Tokyo, Japan) and the Statens Serum Institute (SSI, Copenhagen, Denmark). O genotyping [12] was also performed for O serogroup untypable (OUT) *E. coli.*

### Whole-genome sequencing and bioinformatics analysis of E. coli OUT (OgGp9):H18

Whole-genome sequencing of *E. coli* OUT (OgGp9):H18 using the DNBSEQ-G400 Instrument (MGI Tech, Shenzhen, China) was performed for core genome (cg) multilocus sequence typing (MLST) [13] and single-nucleotide polymorphism (SNP) analysis [14, 15]. The detailed procedures of each experiment and analysis are described in Supplementary material. The presence of the above virulence factors in the genomes of *E. coli* OUT (OgGp9):H18 strain isolated from a milk sample (ESC818) and a patient (ESC828) was searched by the virulence factor database [16] and BLASTN program [17] with default settings.

### Acid tolerance of E. coli OUT (OgGp9):H18

*E. coli* OUT (OgGp9):H18 strains (ESC818 and ESC 828), *E. coli* K-12 (NBRC 3301; National Institute of Technology and Evaluation Biological Resource Center, Chiba, Japan), and EHEC O157:H7 (EC7, a strain from a patient from an outbreak in Sakai City, 1996) [18] cells were cultured in TSB (Oxoid) at 37 °C for 18 h. The cultures were diluted in buffered peptone water (BPW, pH 7.0; Nissui) to 10^6^ CFU/mL. The bacterial dilutions (0.1 mL) were inoculated into each 0.9 mL BPW at pH 2.5, 3.0, 4.0, and 7.0 prepared with 1 N HCl. BPW-inoculated *E. coli* strains were incubated at 37 °C for 3 h. After incubation, the cultures were diluted to 10^−6^ and 10^−7^ in phosphate-buffered saline (PBS), and the dilutions (0.1 mL) were inoculated onto TSA in duplicates. After culturing at 37 °C for 18 h, colonies were counted to confirm the populations in BPW at various pH. The acid tolerance test was performed in triplicates.

## Comparative analysis of acid fitness island

The acid fitness island (AFI) [19] in *E. coli* K-12, *E. coli* OgGp9:H18 (ESC818), and *E. coli* O157:H7 (Sakai) genomes was browsed and extracted using Artemis [20]. Alignments between each locus were generated using the BLASTN program [17] with default settings and then analysed and visualised using Easyfig [21].

### Freezing resistance of E. coli OUT (OgGp9):H18

*E. coli* OUT (OgGp9):H18 strain (ESC818) was cultured in TSB at 37 °C for 18 h, and the culture (40 μL) was inoculated into four tubes containing 40 mL milk purchased in Tokyo. To confirm the number of bacteria inoculated, the bacterial culture was diluted to 10^−6^ and 10^−7^ in PBS, and then, the dilutions (0.1 mL) were inoculated onto TSA in duplicates. After culturing at 37 °C for 18 h, colonies were counted. The inoculated milk samples were stored in a refrigerator at −28 °C. Immediately after and at one, three, and seven days, the milk inoculated with the strain was thawed. The strain population was determined using the most probable number (MPN) method [22]. In brief, 10 mL, 1 mL, and 0.1 mL of milk were inoculated into 10 mL CT-mEC, in triplicates. After incubation at 42 °C for 22 h, the cultures were streaked on CHROMagar STEC and incubated at 37 °C for 22 h. The colonies suspected to be *E. coli* were confirmed as OgGp9 by O genotyping. The freezing resistance test was performed in triplicates.

### Quantification of E. coli OUT (OgGp9):H18 and bacterial population in milk

Nineteen milk samples that tested positive for *E. coli* OUT (OgGp9):H18 contamination were quantitatively assessed for the level of contamination by the pathogen using the MPN method described above. The milk samples were also quantitatively tested to measure the bacterial contamination level by the MPN method, using TSB. After incubation at 37 °C for 18 h, the cultures were observed for turbidity and streaked on TSA to determine bacterial growth. Additionally, a real-time PCR was performed targeting 16S rRNA [23] to estimate the bacterial population. *E. coli* (NBRC3972, NITE Biological Resource Center, National Institute of Technology and Evaluation, Tokyo, Japan) cultures in TSB were serially diluted with PBS to 10^−1^ to 10^−8^. DNA was extracted from the dilutions using a QIAamp DNA Mini Kit (Qiagen, Hilden, Germany) for real-time PCR. A portion (0.1 mL) of the 10^−6^ dilution was plated onto TSA in quintuplets and incubated at 37 °C for 24 h to confirm the number of bacteria. A standard curve was constructed using Ct and CFU values, and the bacterial population in the milk was estimated.

## Results

### Detection of staphylococcal enterotoxins, B. cereus enterotoxin and the emetic toxin (cereulide), and C. perfringens enterotoxin in milk

Although contamination with staphylococcal enterotoxins, *B. cereus* enterotoxin, cereulide, and *C. perfringens* enterotoxin in two milk samples served on 14, 15, 16, and 17 June (two samples each) was tested using commercially available test kits or LC–MS/MS analysis, these toxins were not detected in any of the samples.

### Detection of L. monocytogenes, S. aureus, B. cereus, E. albertii, diarrhoeagenic E. coli, and other E. coli in milk

*L. monocytogenes, S. aureus*, *E. albertii,* and diarrhoeagenic *E. coli* were not detected in any of the milk samples that were tested. Cereulide-producing strains of *B. cereus* were isolated from milk samples initially collected by Toyama City Public Health Center served on 14, 15, and 16 June.

*E. coli* other than the diarrhoeagenic *E. coli* described above were isolated from milk samples served on 16 June and not served on 17 June (one sample each), but not from those served on 14 June (two samples), 15 June (two samples), 16 June (one sample), and not served on 17 June (four samples). The O antigens of most *E. coli* isolates were untypable (OUT) by agglutination testing with anti-serum. The O genotype of OUT strains was typed to OgGp9 composed of O genotypes O17, O44, O73, and O106 [12]. Even though OgGp9 strains were tested for anti-O17, O44, O73, and O106 sera, positive reactions for agglutination were not observed. Because the H antigen detected for OgGp9 strains was H18, the serotype of *E. coli* strain was determined as OUT (OgGp9):H18. *E. coli* OUT (OgGp9):H18 formed mauve and red colonies on CHROMagar STEC and DHL agars, respectively.

### Isolation of E. coli OUT (OgGp9):H18 from milk

*E. coli* OUT (OgGp9):H18 was isolated from the milk samples served on 15 June (50.0%, 6/12 samples from 12 schools) and 16 June (90.9%, 10/11 samples from 11 schools) (Table 1). Thus, *E. coli* OUT (OgGp9):H18 was isolated from milk samples served on both days of the foodborne outbreak at a rate of 69.6% (16/23). From the milk samples scheduled but not served on 17 June, *E. coli* OUT (OgGp9):H18 was isolated (25%; 2/8 samples from three schools) but not from the milk served on 14 June (0%; 0/11 samples from 11 schools).

**Table 1.** Qualitative analysis of *Escherichia coli* OUT (OgGp9):H18 contamination in milk carton served at school lunch

Milk served date	Milk produced date	Served school^a^ of the positive and negative milk cartons for *E. coli* OUT (OgGp9):H18 (number of cartons)		*E. coli* OUT (OgGp9):H18 positive rate (%)
		Positive	Negative	
14 June	11 June	No samples	D (1), E (1), F(1), J (1), K (1), L (1), B or C (2), G or H or I (3)	0/11 (0)
15 June	14 June	A (1), D (1), E (1), J (1), K (1), O (1)	F (1), B or C (2), G or H or I (3)	6/12 (50)
16 June	15 June	A (1), D (1), E (1), F (1), J (1), K (1), B or C (1), G or H or I (3)	B or C (1)	10/11 (90.9)
17 June	16 June	M (1), M or N (1)	A (1), M (4), M or N (1)	2/8 (25)

a A total of 15 schools (A-O).

### Isolation of E. coli OUT (OgGp9):H18 from faeces in patients

Most colonies from 64 patients’ faeces on DHL agar were suspected of *E. coli.* The colonies coincident with *E. coli* biochemical characteristics according to the results of TSI and LIM agars were tested for O and H serotyping and genotyping, and *E. coli* OUT (OgGp9):H18 was isolated from 61 of 64 patients (95%). *E. coli* O18 and O68 were isolated from the three other patients.

### Virulence factors of E. coli OUT (OgGp9):H18 from milk and patients

In the genomes of *E. coli* OUT (OgGp9):H18 from a milk sample (ESC818) and a patient (ESC828), typical virulence factors such as diarrhoeagenic *E. coli* that were tested in milk samples; *stx* and *eae* for EHEC; ST and LT for ETEC; *eae* and *bfpA* for EPEC; *aggR* for EAEC; *astA* for EAST1-producing *E.coli*; and *invE* and *ipaH* for EIEC were not found by the BLAST search.

### Genetic relationship among E. coli isolates from milk and a patient and other representative E. coli strains

**Figure 1.**
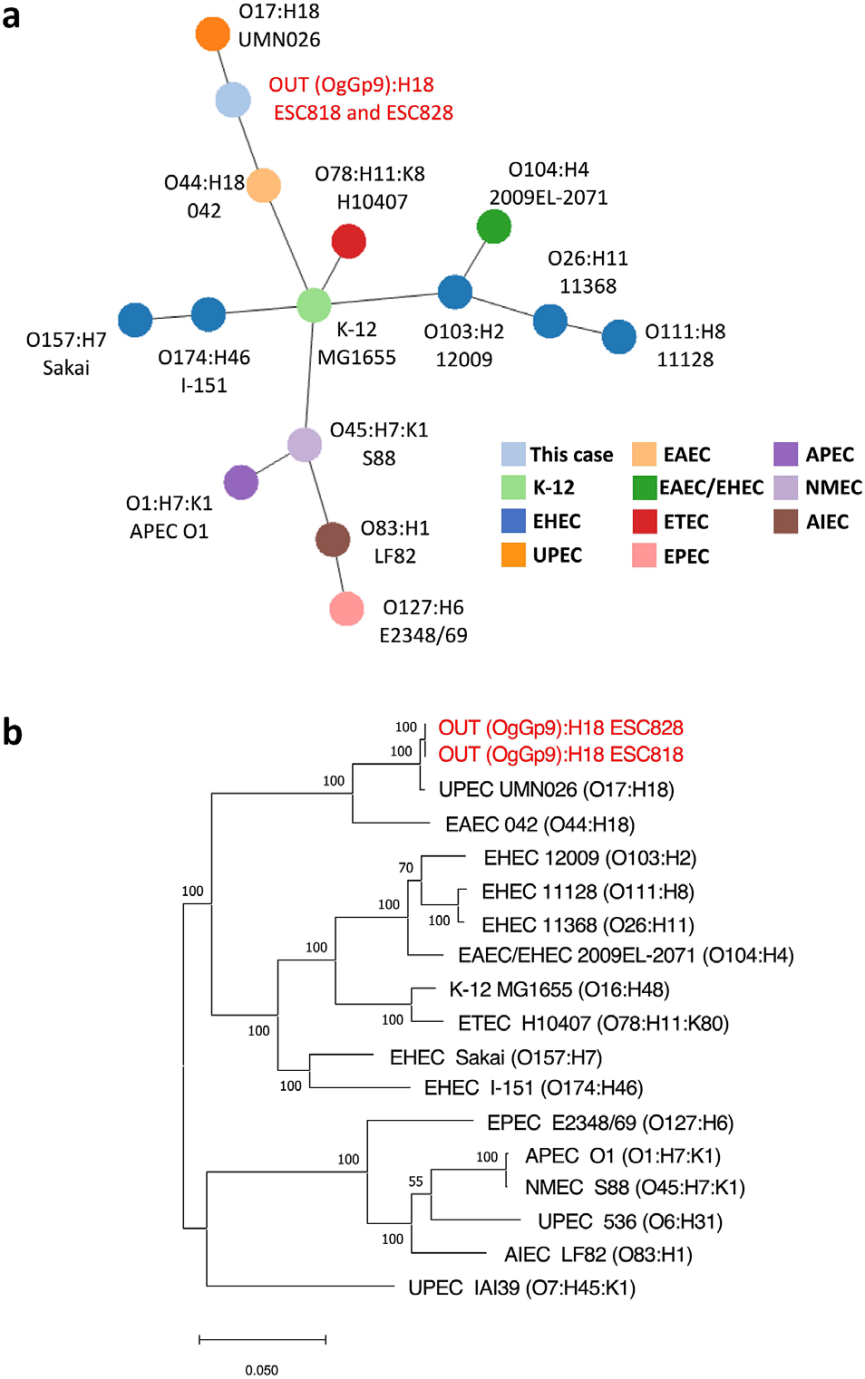
Phylogenetic analysis of OUT (OgGp9):H18 isolates and other *E. coli* strains with serotype and pathotype information. The *E. coli* isolates, in this case, are shown in red. (a) MST based on cgMLST allelic distance of *E. coli* isolates and other strains. The colours of the circles indicate *E. coli* pathotypes. The length between the two circles reflects the genetic distance. (b) Maximum-likelihood phylogenetic tree based on 3,593 SNP sites in the genome backbone of *E. coli* isolates and other strains. The scale bar indicates the number of substitutions per site. K-12: a model strain of *E. coli* (non-pathogenic), EHEC: enterohemorrhagic *E. coli*, UPEC: uropathogenic *E. coli*, EAEC: enteroaggregative *E. coli*, ETEC: enterotoxigenic *E. coli*, EPEC: enteropathogenic *E. coli*, APEC: avian pathogenic *E. coli*, NMEC: neonatal meningitis *E. coli*, AIEC: adherent invasive *E. coli.*

In cgMLST analysis, the genome IDs of all 2,513 loci identified from ESC818 and ESC828 were identical. Additionally, in the cgSNP analysis, only one SNP was detected among the 3,388,601 bp of the core genome in our analysis. These results indicate that isolates from ESC818 and ESC828 were the same clones (Figure 1a,b). Upon conducting the MST analysis based on cgMLST, these isolates were classified into the same branch as uropathogenic *E. coli* O17:H18 (UMN026) and EAEC O44:H18 (042) (Figure 1a). Similarly, the phylogenetic tree based on cgSNP indicated that these isolates were relatively more related to UPEC (UMN026) and EAEC (042) than other strains (Figure 1b).

### Acid tolerance of E. coli OUT (OgGp9):H18

**Figure 2.**
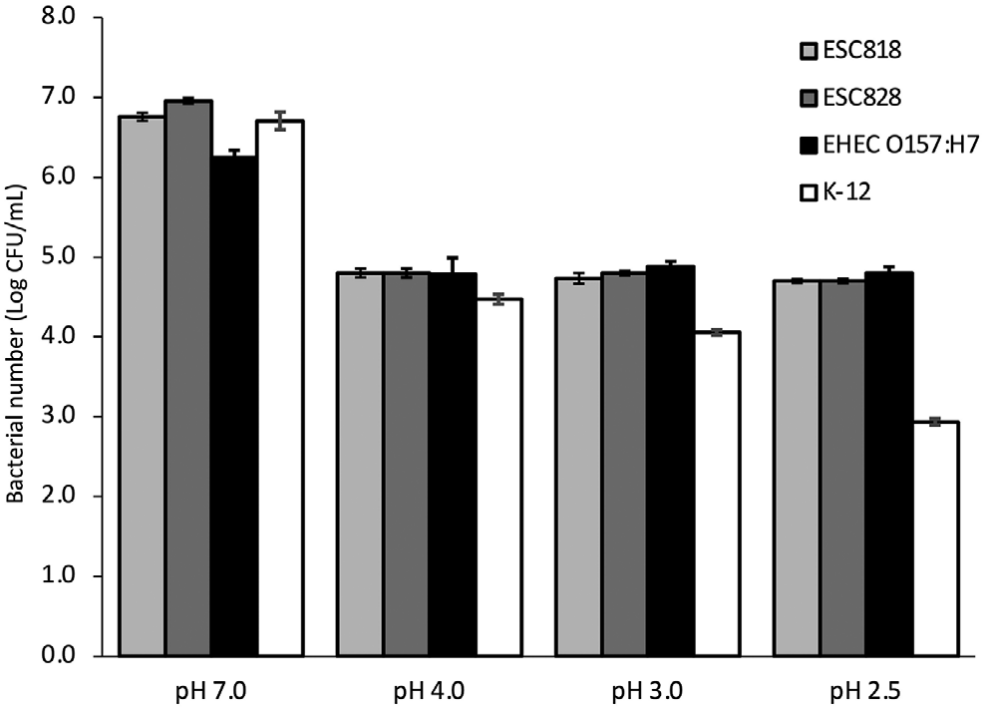
Comparison of acid tolerance of *E. coli* strains at different pH in buffered peptone water. Acid tolerance of ESC818 – *E. coli* OUT (OgGp9):H18 isolated from milk samples, ESC828 *– E. coli* OUT (OgGp9):H18 isolated from patient faeces, EHEC O157:H7 – enterohemorrhagic *E. coli* derived from the foodborne outbreak, and K-12 – a model strain of *E. coli* (non-pathogenic) at pH 2.5, 3.0, 4.0, and 7.0 in buffered peptone water is shown. Error bars indicate standard deviation (*n* = 3).

The mean populations of *E. coli* OUT (OgGp9):H18 strains (ESC818 and ESC 828), EHEC O157:H7 (EC7), and *E. coli* K-12 in BPW at various pH values were 3.9 log CFU/ mL, 4.0 log CFU/ mL, 3.9 log CFU/ mL, and 3.9 log CFU/ mL, respectively. All the strains grew to 6.2–7.0 (ca 6.7) log CFU/mL at pH 7.0 (Figure 2). However, *E. coli* OUT (OgGp9):H18 strains and EHEC O157:H7 grew slightly at pH 2.5, 3.0, and 4.0. In addition, *E. coli* K-12 grew slightly at pH 4.0, although the inoculation level was maintained at pH 3.0 and decreased to 2.9 log CFU/ mL at pH 2.5.

### Comparative analysis of AFI

**Figure 3.**
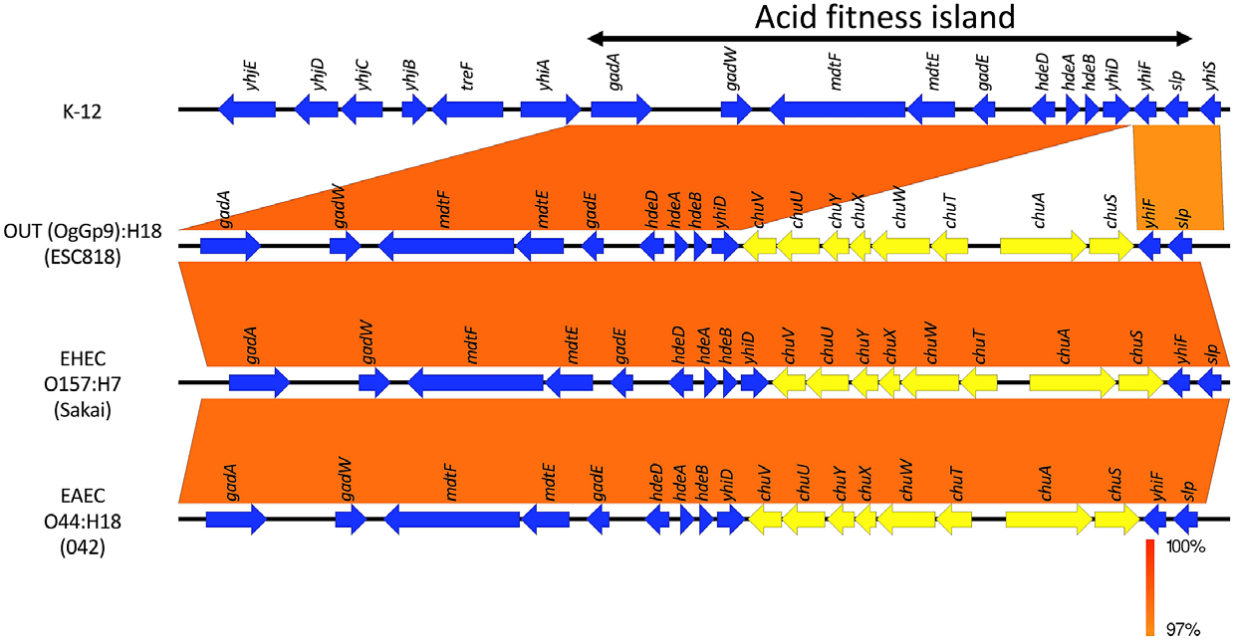
Comparative analysis of the acid fitness island (AFI) in K-12, OgGp9:H18 (ESC818), EHEC O157:H7 (Sakai), and EAEC O44:H18 (042) genomes. Sequence alignment of the AFI (between *gadA* and *slp*) in K-12, OgGp9:H18 (ESC818), EHEC (Sakai), and EAEC (042) genomes is shown. Vertical boxes between each sequence indicate similarity according to BLASTN (red for matches in the same direction). Blue arrows with annotation are coding sequences except for genes in the O-island 140 (inserted between *yhiF* and *yhiD*, yellow arrows).

In the OUT (OgGp9):H18 genome, the AFI, a cluster of genes responsible for acid tolerance of widespread *E.coli* from non-pathogenic K-12 to EHEC, such as *gadA* glutamate decarboxylase [19], was observed (Figure 3). Additionally, the island of the OUT (OgGp9):H18 genome and O-island 140 consist of genes involved in iron uptake, such as *chuA* [24], similar to the O157:H7 genome. O-island 140 was also present in the AFI of the EAEC (042) genome (Figure 3).

### Freezing resistance of E. coli OUT (OgGp9):H18

The mean populations of *E. coli* OUT (OgGp9):H18 in the inoculated milk sample were 413 CFU/40 mL. The mean populations of *E. coli* OUT (OgGp9):H18 were 600, 1,033, 813, and 600 MPN/100 mL after 0, 1, 3, and 7 days of storage, respectively (Table 2). No large decrease was observed during the storage period.

**Table 2.** Survival of *Escherichia coli* OUT(OgGp9):H18 in frozen milk

Freezing period (day)	Bacterial population (MPN/100 mL)
0	600 ± 365^a^
1	1,033 ± 971
3	813 ± 405
7	600 ± 365

a Mean ± SD.

### Quantification of E. coli OUT (OgGp9):H18 and bacterial populations in milk samples

The mean populations of *E. coli* OUT (OgGp9):H18 in six milk samples from six schools served on 15 June, five samples from five schools served on 16 June, and one sample from one school scheduled but not served on 17 June were 8.4 MPN/100 mL, 7.0 MPN/100 mL, and 9.2 MPN/100 mL, respectively (Table 3). The mean bacterial populations in milk samples on 14, 15, 16, and 17 June were 710 MPN/100 mL, 299 MPN/100 mL, 610 MPN/100 mL, and 19,367 MPN/100 mL, respectively. The mean populations were also estimated by the standard curve analysis of real-time PCR targeting 16S rRNA and were 3.8 log CFU/mL, 3.8 log CFU/mL, 5.0 log CFU/mL, and 5.5 log CFU/mL, respectively.

**Table 3.** Quantitative analysis of *Escherichia coli* OUT (OgGp9):H18 contamination in milk carton served in school lunch

Milk served date	Milk produced date	No. of tested milk cartons	*E. coli* OUT (OgGp9):H18						
			No. of positive milk cartons	MPN value (MPN/100 mL)		MPN value of viable bacteria (MPN/100 mL)		Bacterial count by real-time PCR for 16S rRNA (log CFU/mL)	
				Mean	SD	Mean	SD	Mean	SD
14 June	11 June	5	0	NT	NT	710	356	3.8	0.3
15 June	14 June	6	6	8.4	7.5	299	425	3.8	0.8
16 June	15 June	5	5	7.0	3.1	610	461	5.0	0.3
17 June^a^	16 June	3	1	9.2	NA	19,367	17,507	5.5	0.3

a Delivered to schools but not served.

## Discussion

After Toyama City Public Health Center detected the foodborne outbreak, patient samples were immediately analysed at the centre. Tests conducted for many major foodborne bacteria and norovirus were negative. A portion of the milk samples was also analysed at NIHS. Any bacterial toxins and pathogenic bacteria investigated in the study, other than *B. cereus*, were not detected. Although cereulide-producing strains of *B. cereus* were isolated, it was determined that the strains were not related to food poisoning because they were detected in all tested milk samples, including those irrespective of the outbreak. Notably, *E. coli* was predominantly isolated from milk cartons that were served in school lunches on the days that caused the foodborne outbreak, but not in those scheduled to be served on other days. The serotype was determined to be OUT (OgGp9):H18. The major symptoms of the patients such as abdominal pain and diarrhoea were consistent with those of diarrhoeagenic *E. coli* infection. In addition, *E. coli* was detected in most of the faecal samples of patients. The *E. coli* OUT (OgGp9):H18 strain from milk was similar to those from patients, as assessed by cgMLST and cgSNP analyses. These results suggest that the intake of *E. coli* OUT (OgGp9):H18-contaminated milk induced the foodborne outbreak. Additional microbiological tests and epidemiological information indicated that *E. coli* OUT (OgGp9):H18 was the causative bacterium of this outbreak.

Gastrointestinal pathogens must survive the acidic conditions in the stomach to infect the host; thus, acid tolerance is related to virulence [25]. We observed that the acid tolerance of *E. coli* OUT (OgGp9):H18 was similar to that of EHEC O157:H7 (Figure 2). The populations of *E. coli* OUT (OgGp9):H18 strains and EHEC O157:H7 were maintained at 4.7–4.9 log CFU/mL under acidic conditions (pH ranging between 2.5 and 4.0). The acid tolerance of *E. coli* OUT (OgGp9):H18 strain was genetically analysed. Although genes are responsible for acid tolerance in *E. coli* such as *gad* and *hde* cluster in the AFI and are conserved from the non-pathogenic K-12 to EHEC [24], their transcriptomic responses differ at varying pH conditions, resulting in differences in acid tolerances of the *E. coli* strains [26, 27]. In the AFI of the EHEC genome, a *chu* gene cluster, involved in iron uptake, designated O-island 140, is inserted, implying that O-island 140 contributes to the enhanced acid tolerance of EHEC compared with K-12 [24]. Iron is an essential cofactor of several enzymes, and it has been reported that acidic pH enhances the expression of genes involved in iron uptake in K-12 cells [28]. In the above transcriptional studies, the upregulation of EHEC-specific genes involved in iron uptake, such as *chu* genes, was observed in acid-treated EHEC [26, 27]. Additionally, the enhanced expression of *chu* genes along with an acid stress response in EHEC treated with spinach root exudates was observed, but it was not observed in EHEC treated with spinach leaf extracts, which may be attributed to acidic conditions in root exudates [29]. Considering the pathogenicity of EHEC, the upregulation of *chu* genes in O-island 140 along with genes in the AFI in EHEC within human macrophages contributed to the survival of EHEC in macrophages [30]. These reports imply that *chu* genes in the O-island 140 play a vital role in acidic conditions and contribute to acid tolerance and pathogenicity of EHEC. In the OUT (OgGp9):H18 genome, O-island 140 was also inserted within the AFI, similar to the EHEC genome, which suggests genetic and phenotypic features such as acid tolerance and pathogenesis of this strain. *E. coli* OUT (OgGp9):H18 strains can survive in the stomach, similar to EHEC O157:H7, and the ability to survive would be virulent.

The pathogenicity of *E. coli* OUT (OgGp9):H18 has been analysed vigorously in another study. The strain showed adhesive properties in cultured cells and lethality in mice (Hara-Kudo, Y. et al., personal communication), implying that the strain was pathogenic. The strain was phylogenetically closely related to some strains of EAEC (042) and UPEC (UMN026). Previously, EAEC OgGp9:H18 strains harbouring *aggR* were also isolated from patients with gastrointestinal symptoms [31]. However, the *E. coli* OUT (OgGp9):H18 strains in our study did not possess typical EAEC virulence factors such as *aggR*, suggesting the presence of other atypical virulence factors in the strains. The results of microbiological tests and epidemiological information indicate that the *E. coli* OUT (OgGp9):H18 strain was the cause of the outbreak, although the details of the pathogenesis are not clear at present. In another study, the virulence factors of *E. coli* OUT(OgGp9):H18 have been analysed and will be revealed in the future.

We investigated the proportion of contamination in milk cartons. Although some of the samples were tested in this study, it appeared that 50% of the milk cartons served on 15 June were contaminated with *E. coli* OUT (OgGp9):H18 (Table 1). In addition, milk cartons served on 16 June and scheduled but not served on 17 June were also contaminated with *E. coli* OUT (OgGp9):H18 at rates of 90.9% and 25% (Table 1), respectively. It was indicated that contamination of *E. coli* OUT (OgGp9):H18 to milk occurred on 14 June and continued for the next two days. The lack of cleaning of manufacturing lines or sanitary work could be considered one of the causes of this continuous contamination.

Because of the intake of 200 mL milk carton per patient, the ingestion dose of *E. coli* OUT (OgGp9):H18 was estimated at 15–18 based on the quantitative data of pathogen contamination in milk by the MPN method. The milk samples were stored at −20 °C for a few days. Freezing might have affected the survival rate of *E. coli* OUT (OgGp9):H18. Thus, we investigated a decrease in *E. coli* OUT (OgGp9):H18 populations in milk by freezing, and then, they were minimally affected by freezing for 7 days (Table 2). Therefore, the estimated ingestion dose was reasonable. Usually, infectious doses of EHEC have been estimated at less than 10 to several hundred [32, 33] and this trait is attributed to acid tolerance [25]. Since major symptoms were abdominal pain and diarrhoea but not severe such as bloody diarrhoea and haemolytic uraemic syndrome (Suzuki, T. et al., personal communication), the pathogenicity of the *E. coli* OUT (OgGp9):H18 strains in this study was considered to be lower than that of EHEC. However, it is consistent that the infectious dose of the strains is at the same level as EHEC, because the strains can survive at low pH, as same as EHEC.

In this study, viable bacterial populations in milk samples were analysed using the MPN method, and the populations in milk cartons served on 15 June (mean 299 MPN/100 mL) and 16 June (mean 610 MPN/100 mL) were similar to those on 14 June (mean 710 MPN/100 mL) (Table 3). Large differences were not observed among the viable bacterial populations in milk cartons from 14, 15, and 16 June. This indicated that the pasteurisation of milk served on 15 and 16 June was similar to that on 14 June. In addition, it was shown that the bacterial number of *E. coli* OUT (OgGp9):H18 strain decreased by 5 log CFU at 65 °C for 1 min, similar to those of *E. coli* K-12 and EHEC O157:H7, and therefore, heat resistance of the strain was not observed (Hara-Kudo, Y. et al., personal communication). These results indicate that contamination by *E. coli* OUT(OgGp9):H18 might occur after the pasteurisation steps of milk. For example, a surge tank for pasteurised milk or milk packed in cartons would be contaminated. T factory usually produced 6,000–7,000 cartons (200 mL) a day and actually produced 6,851 cartons on 11 June though 7,840 cartons were produced on 14 June. It might lead to some problems.

After the foodborne outbreak, the T milk factory was inspected to identify critical points, leading to *E. coli* OUT (OgGp9):H18 contamination, and measures for preventing such recurrence were recommended by the Toyama City Government and the Ministry of Labour, Health, and Welfare. Although the origin of *E. coli* OUT (OgGp9):H18 and the factors for contamination of milk cartons and continuous contamination were not clarified, it appeared that cross-contamination of pasteurised milk with raw milk by unsanitary handling, insufficient cleaning of raw milk tanks and milk cartons packing equipment, failure on temperature control of pasteurised milk, and structural defect of surge tanks for pasteurised milk were suspected as various aspects with potential risk for bacterial contamination and the growth (Suzuki, T. et al., personal communication). Along with them, the Japan Dairy Industry Association provided technical advice to the T milk factory for improving their manufacturing process.

## Supporting information

Hirose et al. supplementary material Hirose et al. supplementary material

## Data Availability

The draft genomes of *E. coli* OUT (OgGp9):H18 from milk (ESC818) and a patient (ESC828) were deposited at GenBank/EMBL/DDBJ under BioProject number PRJDB15309.

## Supplementary material

For supplementary material accompanying this paper visit http://doi.org/10.1017/S0950268823001395. click here to view supplementary material
