# Supplementary material for: Atypical diarrhoeagenic Escherichia coli in milk related to a large foodborne outbreak
Source: Epidemiol Infect. 2023 Sep 11;151:e150. doi: 10.1017/S0950268823001395 (PMC10540162; doi:10.1017/S0950268823001395)
Supplement: Hirose et al. supplementary material [file S0950268823001395sup001.docx]

**Epidemiology and Infection**

**Atypical diarrheagenic *Escherichia coli* in milk related to a large foodborne outbreak**

Shouhei Hirose^1^, Kenji Ohya^1^, Tomoya Yoshinari^1^, Takahiro Ohnishi^1^, Katsumi Mizukami^2^, Tomikatsu Suzuki^2^, Kenji Takinami^2^, Takayoshi Suzuki^3^, Kenichi Lee^4^, Sunao Iyoda^4^, Yukihiro Akeda^4^, Yuichiro Yahata^5^, Yuuki Tsuchihashi^5^, Tomimasa Sunagawa^5^, Yukiko Hara-Kudo^1,*^

**Supplementary Methods**

**Detection of staphylococcal enterotoxins, *B. cereus* enterotoxin and the emetic toxin (cereulide), and *C. perfringens* enterotoxin** **in milk**

A total of 11 milk samples; two milk samples from two schools served on June 14^th^ suggested no relation with the food poisoning by epidemiological investigation, two milk samples from two schools on June 15^th^, two milk samples from two schools on June 16^th^ suggested the relations, and five milk samples from one school stored in the schools for lunches on June 17^th^ but not served, were tested for investigation on bacterial toxins. Additionally, five milk samples from one school stored for lunches on June 17^th^, but not served, were tested for the presence of bacterial toxins. Aliquots of 50 mL, 0.1 mL, and 0.1 mL milk samples were used for analyses of staphylococcal enterotoxins by VIDAS Staph enterotoxin II kit (BioMerieux, France), *B. cereus* enterotoxin by reversed passive latex agglutination kits (CRET-RPLA, Denka Company Limited, Tokyo, Japan) and *C. perfringens* enterotoxin by PET-RPLA (Denka), respectively. Each test was performed in accordance with the manufacturer’s instructions. Cereulide content in each milk sample (0.5 mL) was quantified by liquid chromatography-tandem mass spectrometry (LC-MS/MS), and toxin extraction was done according to a previously reported method [34].

**Detection of *L. monocytogenes*, *S. aureus,* *B. cereus*, *Escherichia albertii,* and diarrheagenic** ***E. coli* in milk**

The milk samples described above were also tested to investigate other foodborne pathogens.

For *L. monocytogenes*, the samples were cultured in half-Fraser broth at 30 °C, followed by Fraser broth at 37 °C for selective enrichment. After enrichment, each culture was streaked onto two kinds of selective agar, CHROMagar Listeria (CHROMagar, Paris, France) and Oxford agar (Oxoid, Basingstoke, UK), for the isolation of *L. monocytogenes*.

For *S. aureus* and *B. cereus,* the milk samples were cultured in tryptic soy broth (TBS; Oxoid) with 7.5% NaCl at 37 °C. After enrichment, each culture was streaked on mannitol salt agar (Nissui, Tokyo, Japan) and incubated at 37 °C. The isolates were identified using matrix-assisted laser desorption/ionization time-of-flight mass spectrometry and the SARAMIS system (Shimadzu, Kyoto, Japan). The isolated *B. cereus* strains were inoculated with store-purchased milk and incubated at 32 °C. After enrichment, cereulide in each culture was quantified by LC-MS/MS, as described above.

In addition, the milk samples (25 g) were cultured in 225 mL of modified *E. coli* broth (mEC, Nissui) or mEC supplemented with cefixime and tellurite (Oxoid) (CT-mEC) at 42 °C to detect *E. albertii* and diarrheagenic *E. coli*: enterohemorrhagic *E. coli* (Shiga-toxin producing *E. coli,* STEC), enterotoxigenic *E. coli* (ETEC), enteroinvasive *E. coli* (EIEC), enteropathogenic *E. coli* (EPEC) , enteroaggregative *E. coli* (EAEC) and Enteroaggregative *E. coli* heat-stable enterotoxin 1 (EAST1) producing *E. coli*. DNA was extracted from the cultures using the alkaline heat extraction method [35]. *E.* *albertii*-specific real-time PCR and PCR assays pathogenic factors genes of diarrheagenic *E. coli* were performed.

**Whole genome sequencing of** ***E. coli* OUT (OgGp9):H18**

The genomic DNA of *E. coli* OUT (OgGp9):H18 strains from milk (ESC818) and a patient (ESC828) was extracted using the blood and cell culture DNA Midi kit (Qiagen, Hilden, Germany). Genomic DNA libraries were prepared using the MGIEasy FS DNA Library Prep Set (MGI Tech, Shenzhen, China). Paired-end sequencing (2 × 200 bp) was performed using the DNBSEQ-G400 High-throughput Sequencing Set on the DNBSEQ-G400 Instrument (MGI Tech). The coverage obtained for the ESC818 and ESC828 genomes was x211 and x185, respectively. The raw reads were trimmed using cutadapt version 1.9.1 [36] and were quality filtered (threshold quality value set at 20) by sickle version 1.33 [37]. The genome was assembled using Spades version 3.13.2 [38] and annotated using the DDBJ Fast Annotation and Submission Tool [39].

**Core genome (cg) multilocus sequence typing (MLST) and single nucleotide polymorphism (SNP) analysis of *E. coli* OUT (OgGp9):H18**

The cgMLST comparison was performed following the EnteroBase protocol (https://enterobase.warwick.ac.uk/species/index/ecoli) [13]. The identifier (ID) of each locus (2513 loci) was assigned by loading the assembled reads of the ESC818 and ESC828 genomes, and genome data of representative *E. coli* OUT (OgGp9):H18 strains retrieved from NCBI into the EnteroBase database. A minimum spanning tree (MST) of the cgMLST profile was built by analyzing the allelic matrix using PHYLOViZ Online [40]. The cgSNPs of the ESC818 and ESC828 contigs and the genome of representative *E. coli* strains were extracted using BactSNP version 1.1.0 [14] with the genome of *E. coli* O157:H7 Sakai (GenBank accession No.: BA000007.3) as a reference. The recombinogenic regions were detected and removed by Gubbins version 2.4.1 [41].Repetitive regions longer than 50 bp were detected by MUMmer v.4.0.0rc1 (nucmer, repeat-match, and exact-tandems functions) [42] and removed for further analyses, as were prophage regions. Finally, 30,828 SNP sites from 3,388,601 bp of conserved backbone were used for further analyses. A phylogenetic tree was reconstructed using the maximum likelihood method with 1000 bootstraps by using IQTREE version 2.0.3 [43].

**Supplementary References**

34. **Koike H*, et al.*** (2018) Identification and quantification of cereulide in cow's milk using liquid chromatography-tandem mass spectrometry. *Food Additives & Contaminants;* **35**: 2424-2433.

35. **Hara-Kudo Y*, et al.*** (2016) An interlaboratory study on efficient detection of Shiga toxin-producing *Escherichia coli* O26, O103, O111, O121, O145, and O157 in food using real-time PCR assay and chromogenic agar. *International Journal of Food Microbiology;* **230**: 81-88.

36. **Martin M.** (2011) Cutadapt removes adapter sequences from high-throughput sequencing reads. *EMBnet journal;* **17**.

37. **Joshi NA FJS.** (2011) A sliding-window, adaptive, quality-based trimming tool for FastQ files (Version 1.33). [software]. *Available from https://github.com/najoshi/sickle [Last accessed: 03/16/2023]*.

38. **Prjibelski A*, et al.*** (2020) Using SPAdes De Novo Assembler. *Current Protocols in Bioinformatics;* **70**: e102.

39. **Tanizawa Y, Fujisawa T, Nakamura Y.** (2018) DFAST: a flexible prokaryotic genome annotation pipeline for faster genome publication. *Bioinformatics;* **34**: 1037-1039.

40. **Ribeiro-Goncalves B*, et al.*** (2016) PHYLOViZ Online: web-based tool for visualization, phylogenetic inference, analysis and sharing of minimum spanning trees. *Nucleic Acids Research;* **44**: W246-W251.

41. **Croucher NJ*, et al.*** (2015) Rapid phylogenetic analysis of large samples of recombinant bacterial whole genome sequences using Gubbins. *Nucleic Acids Research*; **43**: e15.

42. **Marcais G*, et al.*** (2018) MUMmer4: A fast and versatile genome alignment system. *PLOS Computational Biology*; **14**: e1005944.

43. **Nguyen LT*, et al.*** (2015) IQ-TREE: a fast and effective stochastic algorithm for estimating maximum-likelihood phylogenies. *Molecular Biology and Evolution*; **32**: 268-274.
